# Safety and tolerability of frozen, capsulized autologous faecal microbiota transplantation. A randomized double blinded phase I clinical trial

**DOI:** 10.1371/journal.pone.0292132

**Published:** 2023-09-27

**Authors:** Måns Stefansson, Oscar Bladh, Ola Flink, Otto Skolling, Hans-Peter Ekre, Lars Rombo, Lars Engstrand, Johan Ursing

**Affiliations:** 1 Centre for Clinical Research Sörmland, Uppsala University, Eskilstuna, Sweden; 2 Department of Clinical Sciences Danderyd Hospital, Karolinska Institutet, Stockholm, Sweden; 3 Department of Infectious Diseases, Danderyd Hospital, Stockholm, Sweden; 4 Bactaviva AB, Stockholm, Sweden; 5 Centre for Translational Microbiome Research, Department of Microbiology, Tumor and Cell Biology, Karolinska Institutet, Solna, Sweden; Charite Universitatsmedizin Berlin, GERMANY

## Abstract

**Background:**

Faecal microbiota transplantation (FMT) is recommended treatment for recurrent *Clostridioides difficile* infection and is studied as a potential modifier of other gastrointestinal and systemic disorders. Autologous FMT limits the potential risks of donor transplant material and enables prophylactic treatment. Capsulized FMT is convenient and accessible, but safety data are lacking.

**Aims:**

To describe safety and tolerability of capsules containing autologous FMT, compared to placebo, in healthy volunteers treated with antibiotics.

**Method:**

Healthy volunteers without antibiotic exposure during the past three months, that had a negative *Clostridioides difficile* stool sample, were recruited. Study persons donated faeces for production of capsules containing autologous microbiota. They were then given Clindamycin for seven days to disrupt the intestinal microbiota, which was followed by a two-day washout. Study persons were then randomized (1:1) to unsupervised treatment with autologous faecal matter or placebo, with two capsules twice daily for five days. A standardized questionnaire about side effects and tolerability, daily until day 28, and on days 60 and 180, was completed.

**Results:**

Twenty-four study persons were included, all completed the treatment. One person from the placebo and FMT groups each, were lost to follow up from days 21 and 60, respectively. No study person experienced serious side effects, but severe fatigue was reported during the antibiotic period (n = 2). Reported side effects were mild to moderate and there were no significant differences between the groups. Reported general and intestinal health improved significantly and similarly in both groups after the antibiotic treatment. Time to normalized intestinal habits were 17 and 19 days from study start in the placebo group and the FMT group, respectively (p = 0.8).

**Conclusion:**

Capsulized frozen autologous faecal microbiota transplantation was safe and well tolerated but did not affect time to normalized intestinal habits compared to placebo.

**Trial registration:**

EudraCT **2017-002418-30.**

## Introduction/ Background

The human intestinal microbiota is a complex mixture of bacteria, fungi, protozoa, and virus that interact with each other and the host. The microbiota plays a fundamental role in the development of the immune system and defends the host against infection by competitive colonization [1–4]. Disruption of the microbiota (dysbiosis) has been associated with enteric infections and other gastrointestinal and systemic disorders [2, 3, 5, 6]. The most well-documented effect of antibiotic induced dysbiosis is *Clostridioides difficile* infection [7]. Antibiotic induced dysbiosis has also been shown to select for highly resistant intestinal bacteria up to two years after treatment and to affect the response to novel cancer drugs [8, 9]. There is consequently considerable interest in the possibilities of reconstituting or modulating the microbiota.

Restoration of the microbiota through faecal microbiota transplantation (FMT) is a recommended treatment for recurrent *C*. *difficile* infection [10–13]. FMT also resulted in decolonization of antibiotic resistant pathogens in patients with haematological malignancies [14, 15]. However, FMT is not routinely available and only approximately 10% of eligible patients received FMT for recurrent *C*. *difficile* infection in Stockholm County in 2018 (unpublished data). The main obstacles are identification of donors, the complexity of FMT administration via colonoscopy or gastroscopy, and the process of capsulizing faecal samples [16]. Moreover, though FMT is considered safe, there is a risk of transmitting pathogenic organisms and the long-term effects of heterogenic microbiota on the recipient are not known [17].

Replacing donors with autologous faeces circumvents some of these difficulties [18]. Published data on autologous FMT (auto-FMT) are limited and somewhat disparate. Auto-FMT to healthy volunteers, administered via enema, after a short course of amoxicillin-clavulanic acid did not appear to affect the speed of microbiota restoration compared to placebo [19]. However, auto-FMT resulted in rapid microbiota restoration in antibiotic treated patients undergoing allogenic hematopoietic stem cell transplantation [18]. Auto-FMT also resulted in rapid reconstitution of antibiotic induced dysbiosis compared to no treatment or treatment with probiotics in healthy volunteers [20]. Thus, auto-FMT possibly restores antibiotic induced dysbiosis thereby reducing the risk of *C*. *difficile* infection and the burden of multidrug resistant bacteria.

The low risk of transmitting pathogens makes capsulized auto-FMT a suitable candidate for prophylactic treatment, but safety data are lacking. The primary aim of this study was to determine the safety and tolerability of oral, capsulized, frozen auto-FMT in healthy volunteers after antibiotic treatment.

## Material and methods

### Study site and population

The study was conducted at the Department of Infectious Diseases, Danderyd Hospital, Stockholm Sweden between 12^th^ of February 2018 and 19^th^ of December 2018. The study persons were recruited after screening by telephone calls, followed by inclusion visits, between 13^h^ of March until 7^th^ of May 2018. Healthy volunteers were identified by advertisements in the Karolinska Trial Alliance and Studentkanin databases[21, 22]. These sites manage registers of individuals that have reported an interest in participating in research projects.

Mälarsjukhuset in Eskilstuna was a planned second site for conducting the study that was not used for logistical reasons and because enough volunteers were rapidly found at Danderyd hospital.

### Study design

This was a 1:1 randomized, double blinded phase I safety and tolerability study of auto-FMT using acid-resistant capsules compared to placebo capsules. Following donation of a faecal sample, the intestinal microbiota of participants (N = 24) was disrupted by intake of clindamycin capsules. After a two-day washout, study persons were randomized to treatment with autologous faecal matter or placebo. Faecal samples were collected on days 1, 10, 15, 28, 60 and 180 for future microbiome analyses using next generation sequencing, though this data is not yet available. The study protocol is available as S1 File. Clindamycin was chosen to have a clinically relevant model that is known to have a long-term effect on the intestinal microbiome but still safe and well tolerated [23].

Inclusion criteria were as follows; age 18–40 years, ability to swallow study capsules, negative pregnancy test at inclusion, use of an approved method to prevent pregnancy during the study period and written informed consent.

Exclusion criteria were previous or current bowel disorder, delayed gastric emptying syndrome, recurrent aspirations, swallowing dysfunction, antibiotic treatment during the previous three months, regular intake of any medication (except vitamin pills and dietary supplements), body mass index <18.5 and >30, any other significant medical history (except resolved traumatic injury) and identification of *C*. *difficile* in a stool sample prior to study start. Before enrolment in the study, study persons underwent a physical examination. We used the CONSORT checklist when writing this report.

### Ethical permission and registration

The study was conducted in accordance with the Declaration of Helsinki and Good Clinical Practice. Written informed consent was obtained from all participants. Ethical approval was obtained from the regional ethical review board in Stockholm, Sweden (2017/1815-31). The study was registered with EudraCT number 2017-002418-30. The Swedish Medical Products Agency concluded that their approval of the study was not required as clindamycin was not used as an investigational medical product and the autologous faecal matter could not be categorized as a medical product. Since the faecal samples were collected at home for research purposes, the biobank regulations were not applicable, and the samples did not need to be registered in a biobank.

### Randomization and blinding

Randomization was 1:1 with twelve persons in each group. Randomization was done by drawing a folded paper with treatment allocation (12 placebo and 12 study capsule), from a sealed envelope. The paper slips with treatment allocation and sealed envelope were made by a member of the laboratory staff prior to study start. Randomization was done by the laboratory staff that manufactured placebo or study capsules. The laboratory was located several kilometres from the hospital and there was no direct contact between laboratory and hospital study staff.

Identical white capsules were delivered in sealed white plastic containers labelled with the study participants name and study id. Peppermint oil was added to the inside lid of the containers to ensure a non-revealing smell.

### Outcomes

The primary outcome was safety and tolerability by day 28.

The secondary outcomes were time to normalized intestinal habits and time to normalization of intestinal microbiota up to 180 days after study start.

### Procedures

Study persons were seen prior to study start and then on days 1, 10 and 15. At each visit the study persons were examined and questioned about adverse events. All study persons received clindamycin capsules and were prescribed 300 mg three times daily on days 1–7. This was followed by a washout period (days 8 and 9) and treatment with two study capsules, containing microbiota or placebo, twice daily on days 10–14. The study capsules were collected at the hospital, where the first dose was taken under supervision, and then transported home in a cooling box where they were stored in the household freezer until taken.

Study persons completed an electronic questionnaire on days 1–28 (daily), 60 and 180. The number of defecations and their Bristol stool chart score were recorded daily. General and gastrointestinal well-being was also graded on a scale of 1 to 10, with 1 being the lowest and 10 being “best possible health for you”. The experience of the treatment was graded 1 to 5 where 1 corresponded to unproblematic experience and 5 was very unpleasant. Symptoms of nausea, diarrhea, tiredness, vomiting, headache, distended abdomen, abdominal pain, vertigo, rashes, and swallowing difficulties were asked for and graded as 0 (no problem), 1 (minor), 2 (moderate, does not impair daily life), 3 (severe, very undesirable, disruptive symptoms) to 4 (serious or potentially life threatening). Possible adverse events were elicited using a modification of the Common Terminology Criteria for Adverse Events version 3.0 [24]. Study persons recorded daily morning temperatures in the questionnaires using thermometers provided by the study personnel.

### Capsule production

The study persons provided a fresh faecal sample prior to study start. Sixty grams of the faecal sample was diluted with twice the amount of glycerol by weight, homogenized and filtered through a course sieve before freezing to -80°C in Falcon tubes. The procedure was finished within three hours after delivery of faeces.

For capsulation, the frozen sample was thawed in room temperature for two hours, vortexed and then centrifuged at 300 g for 20 minutes. The supernatant was transferred to new Falcon tubes and was centrifuged at 4600 g for 40 minutes. The supernatant was then discarded, and the pellet was weighed and homogenized with the same amount of glycerol. Ten percent of olive oil and 1% of Tween 80 by weight were then added and the mixture homogenized. The mixture was added to white opaque, HPMC acid-resistant capsules nr 0, 0,6 mL (DRcaps™, Capsugel Corp.). The capsules were put into a second slightly larger capsule no 00 to avoid leakage of fecal material and to limit odor.

Twenty capsules were packed in a plastic screw-top container and one drop of peppermint oil was placed on the inside of the lid. The containers were individually labeled for each study person and stored at -20°C until delivery to the subject. Reference samples were stored at -20°C.

Placebo consisted of size 0 capsules filled with cocoa powder mixed in olive oil, placed inside size 00 capsules and packed and stored as study capsules.

### Treatment

Two frozen capsules containing autologous faecal matter or placebo were taken twice daily for 5 days (days 10 to 14), i.e. 20 capsules in total.

### Adverse events

An adverse event was defined as any untoward medical occurrence in a study person who received treatment. Adverse events were graded for: seriousness, intensity and causality and the relationship to the treatment was classified as unlikely, possible, or probable.

### Statistics

No power calculation was done for this safety and tolerability study. Twelve study persons in each arm were considered sufficient to detect major adverse events whilst avoiding unnecessary exposures.

Continuous variables are presented as medians and interquartile ranges (IQR). A gastrointestinal score was calculated by adding the Bristol stool score for each defecation each day. I.e., a person who defecated twice with Bristol stool score of 4 and 7, respectively had a total score of 11 for that day. The time taken from start of clindamycin treatment until the gastrointestinal score stabilised was assessed for each individual patient graphically without prior knowledge of which group the patient belonged to. The time taken until the gastrointestinal score stabilised was assessed in a survival analysis and cox regression. Included time was from start of clindamycin treatment until day 28. The proportional hazard assumption in the cox regression was assessed graphically. The equality of survival functions was compared using the Log Rank Test.

The effect of stopping clindamycin, time and treatment on gastrointestinal score, general and intestinal well-being were assessed by median regression, using a piecewise linear function of time to allow for a different time trend before and after stopping clindamycin treatment. The model included individual fixed effects to capture the between-subject variability. Fischer’s exact test was used to assess differences between the groups regarding adverse events. Study persons with adverse events were pooled irrespective of severity for comparison with patients without adverse events for this analysis. Statistical analyses were performed with STATA version 17 (StataCorp, TX). A *P-*value of < 0.05 was considered significant.

## Results

**Fig 1:**
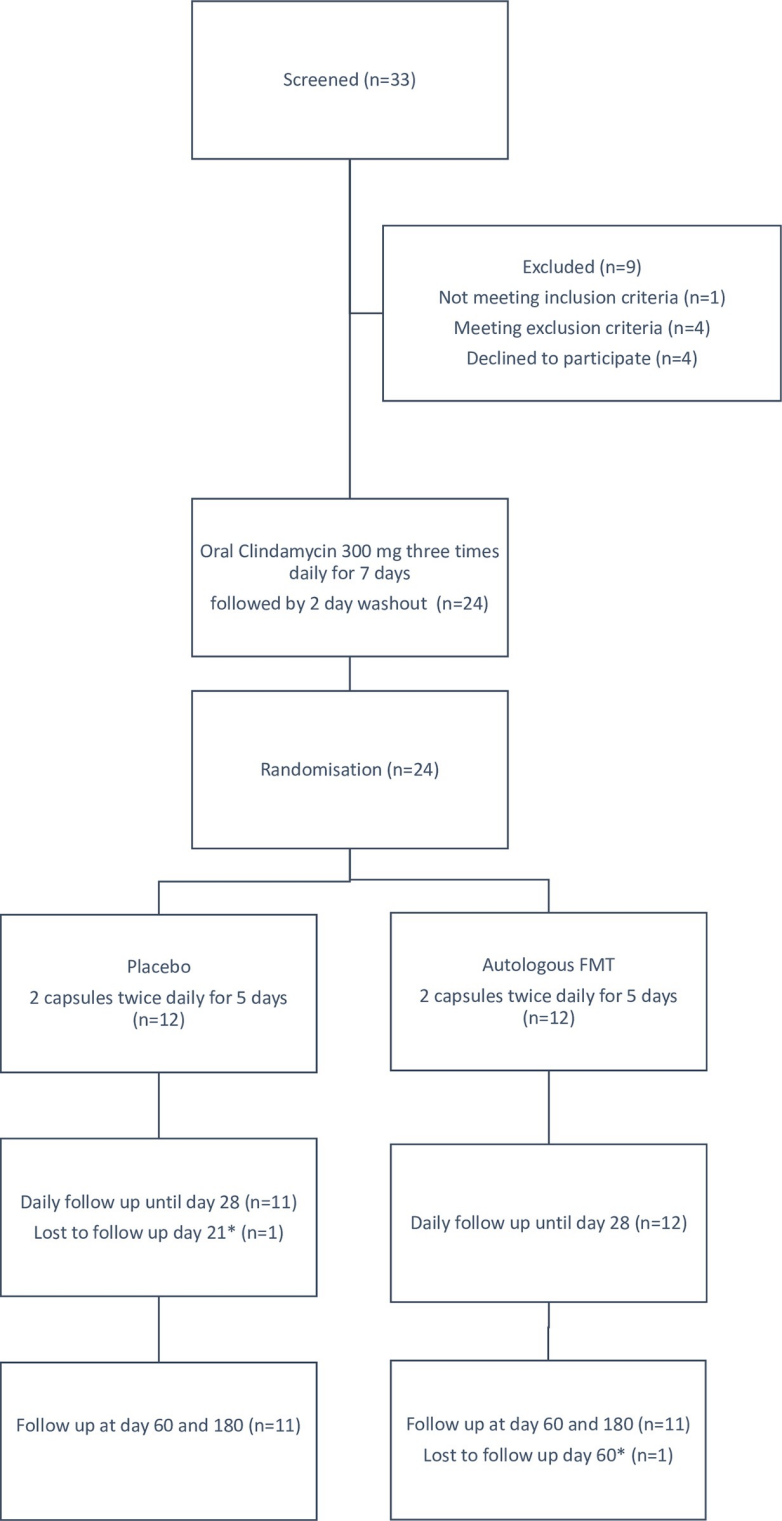
Flowchart for study population.

A total of 33 persons were screened for participation in the study, nine were excluded for the following reasons: not being able to adhere to contraception (N = 1), antibiotic treatment during the last 3 months (N = 1), history of bloody stools (N = 1), undergone several operations (N = 1), possible irritable bowel syndrome (N = 1) and withdrawal of consent (N = 4) (Fig 1).

The characteristics of the included volunteers are described in Table 1.

**Table 1:** Baseline demographic data.

	All	Auto FMT	Placebo
**Number**	24	12	12
**Age (IQR)**	28 (25–35)	29 (26–35)	26 (22–32)
**Female:male**	12:12	7:5	5:7
**BMI (IQR)**	24 (21–26)	24 (21–27)	24 (22–26)
**Smoker**	0	0	0
**Vegetarian**	3	2	1
**Lactose free diet**	2	1	1

IQR = interquartile range

Other than contraceptive pills, the only frequent medications were analgesics without prescription and vitamin supplements.

### Loss to follow up

One person in the placebo group stopped answering the daily questionnaire from day 21 and did not provide a faecal sample for days 28, 60 and 180. Another study person in the auto-FMT group did not send the faecal sample or complete the questionnaire at days 60 and 180.

### Self-reported treatment experience

During treatment with clindamycin the median treatment experience was graded as 1 (range 1–3) and 1 (range 1–3) for the treatment and placebo groups, respectively. The median treatment experience was graded as 1 (range 1–2) and 1 (range 1–2) for the auto-FMT and placebo groups, respectively during intake of study capsules.

### Self-reported general and gastrointestinal health

**Fig 2:**
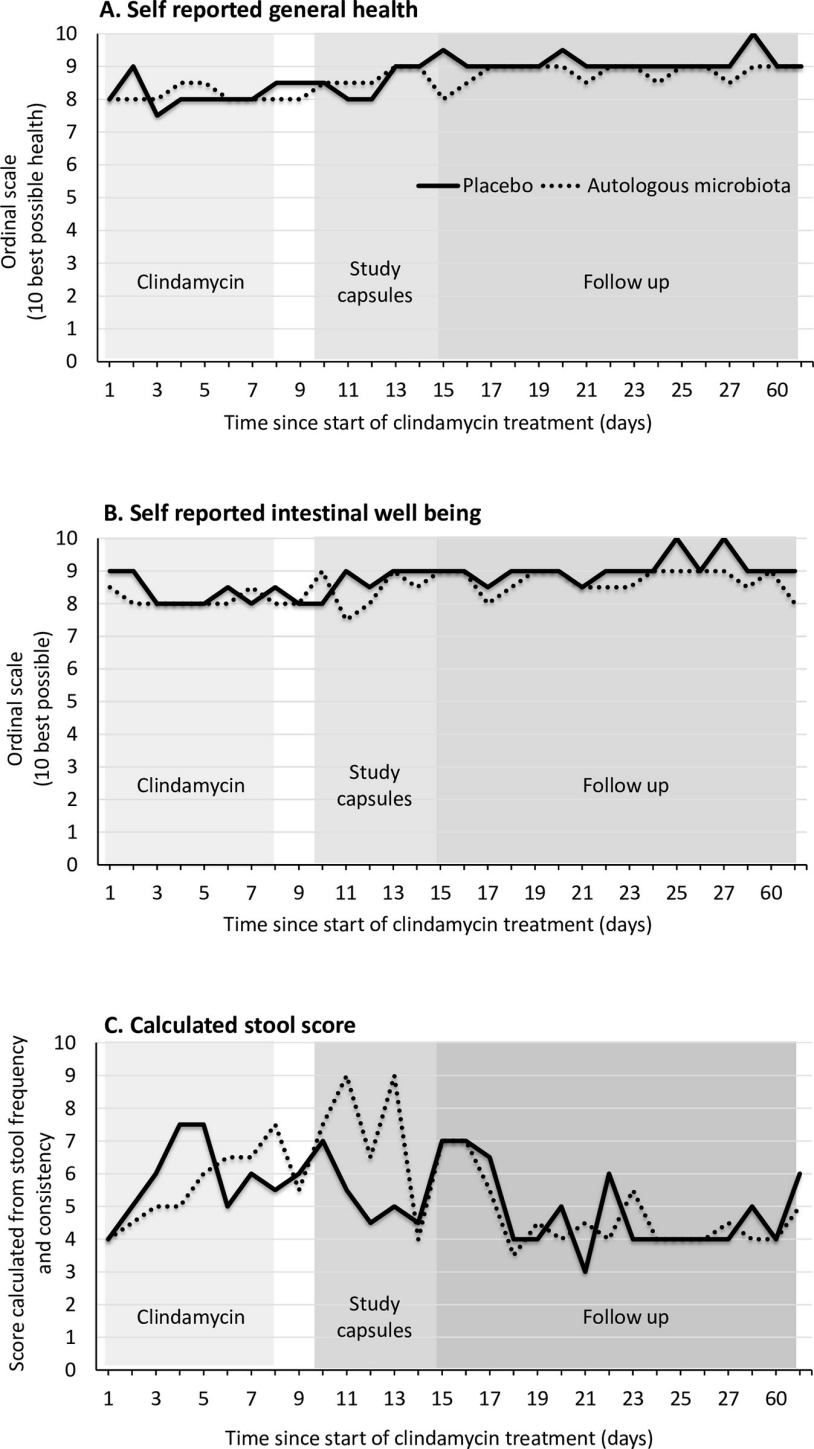
Median self-reported general health (Panel A), intestinal well-being (Panel B), and stool scores (Panel C). From start of clindamycin treatment to end of study. Placebo group (solid line) and autologous faecal microbiota group (dotted line) in panels A, B and C. The stool score was calculated by adding the Bristol stool score for each defecation on each day.

Scores for general health and intestinal well-being were very good throughout the study as shown in Fig 2. General health appeared to be slightly lower during treatment with clindamycin and improved significantly with time when assessed in a univariate fixed effect quantile regression (p<0.001). Intestinal well-being similarly appeared to be lower during treatment with clindamycin and to improve with time, but the finding was not significant (p = 0.07).

### Bristol stool chart and defecations

The calculated stool score is shown in Fig 2 panel C. The score was higher representing more frequent and / or looser stool consistency during treatment with clindamycin. Stopping clindamycin treatment was associated with a decreasing stool score (p = 0.02). Time (p = 0.09) and treatment with placebo versus auto-FMT (p = 0.77) were not significantly associated with the stool score.

### Time to stable intestinal habits

**Fig 3:**
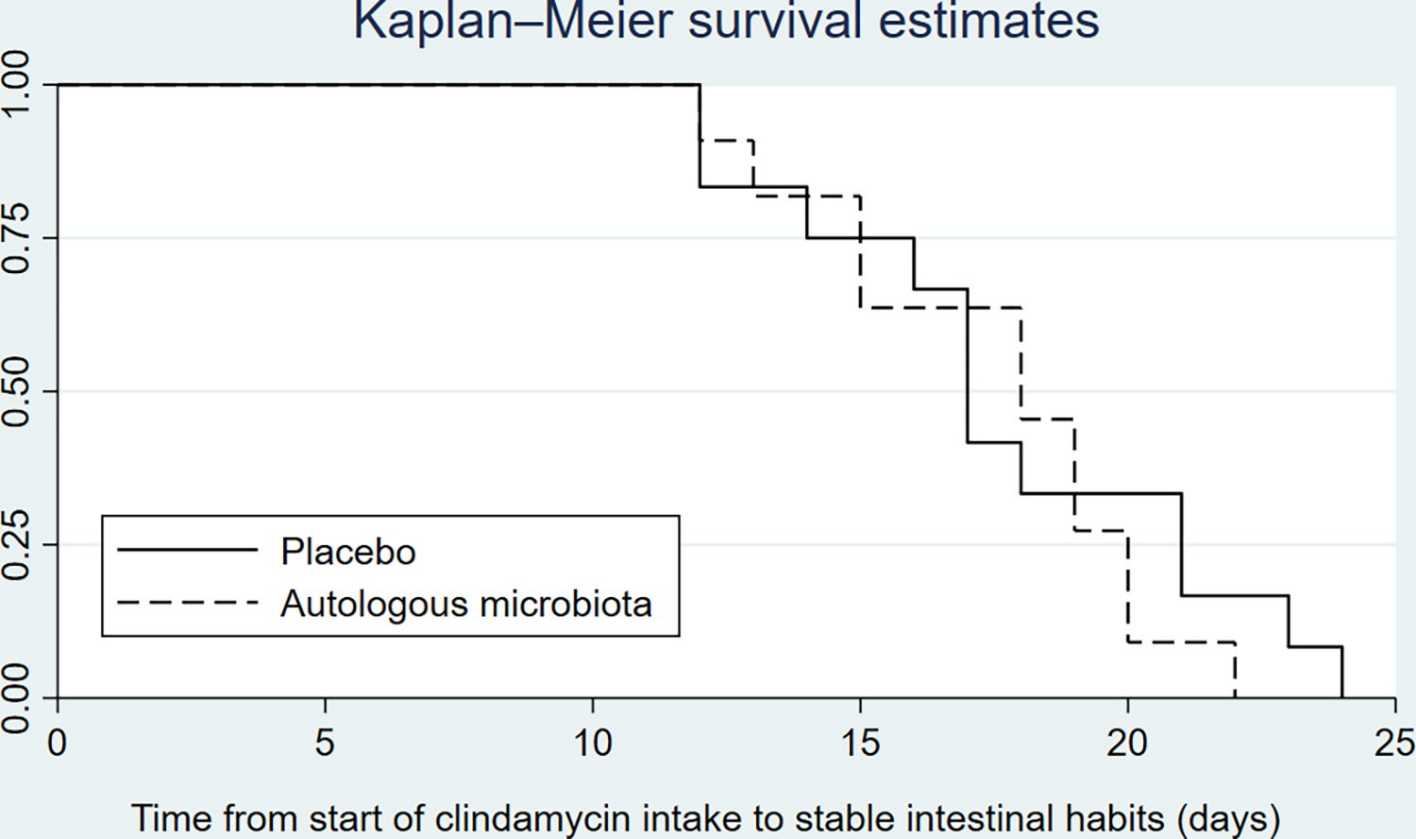
Kaplan-Meier survival estimates. Time to self-reported normalized intestinal stool habits from start of clindamycin treatment.

Time to stable stool habits is shown in Fig 3. The median time taken to reach stable intestinal habits was 17 (95% CI 16–21) days for the placebo group and 19 (95% CI 15–20) days for the auto-FMT group. There was no significant difference between groups, Cox proportional hazard ratio 0.9 (95% CI 0.39–2.06, p = 0.8).

### Specific reported or elicited adverse events

Adverse events are shown in Table 2. Two study persons reported serious fatigue at grade three during clindamycin treatment. The reported degree of fatigue was considered as possibly causally related to the given treatment. The seriousness in these cases was deemed low because the affected persons did not seek medical care, nor did they inform the study staff. A total of thirteen study persons reported fatigue as an adverse event during the first 7 days, eleven of them still had fatigue grade 1–2 during days 10–14 and nine continued to have fatigue during the follow-up period, with a similar proportion in the two groups. Nine subjects reported diarrhea as an adverse event during clindamycin treatment and four reported it during the capsule treatment (three study persons in the auto-FMT group and one in the placebo group). Clindamycin and study capsules containing auto-FMT were classified as probable causes of diarrhea. The intensity in these cases was moderate and the seriousness was considered low. All other reported adverse events in both groups were minor to moderate without significant differences (p = >0,05) between placebo or treatment group. One volunteer had a cold for 5 days without fever. No one reported a daily temperature higher than 38 degrees Celsius.

**Table 2:** Adverse events.

Adverse event score*		Clindamycin Days 1–7^†^				Study capsule Days 10–14^†^			Follow-up Days 15–28^†^		
		0	1	2	3	0	1	2	0	1	2
**Vertigo**	aFMT	11 (92%)	1	0	0	11 (92%)	1	0	12 (100%)	0	0
	Placebo	9 (75%)	2	1	0	12 (100%)	0	0	11 (92%)	1	0
**Nausea**	aFMT	9 (75%)	3	0	0	9 (75%)	3	0	9 (75%)	3	0
	Placebo	7 (58%)	4	1	0	9 (75%)	3	0	11 (92%)	1	0
**Difficulties swallowing**	aFMT	11 (92%)	1	0	0	12 (100%)	0	0	12 (100%)	0	0
	Placebo	9 (75%)	2	1	0	12 (100%)	0	0	12 (100%)	0	0
**Vomiting**	aFMT	10 (83%)	2	0	0	12 (100%)	0	0	12 (100%)	0	0
	Placebo	10 (83%)	1	1	0	12 (100%)	0	0	11 (92%)	1	0
**Distended abdomen**	aFMT	5 (42%)	6	1	0	7 (58%)	3	2	9 (75%)	2	1
	Placebo	8 (67%)	3	1	0	9 (75%)	2	1	10 (83%)	2	0
**Abdominal pain**	aFMT	9 (75%)	3	0	0	10 (83%)	2	0	9 (75%)	2	1
	Placebo	9 (75%)	2	1	0	10 (83%)	1	1	12 (100%)	0	0
**Diarrhoea**	aFMT	6 (50%)	5	1	0	9 (75%)	2	1	8 (67%)	3	1
	Placebo	9 (75%)	2	1	0	11 (92%)	0	1	9 (75%)	3	0
**Headache**	aFMT	8 (67%)	4	0	0	10 (83%)	2	0	8 (67%)	2	2
	Placebo	4 (33%)	6	2	0	8 (67%)	3	1	9 (75%)	3	0
**Fatigue**	aFMT	6 (50%)	4	1	1	6 (50%)	6	0	6 (50%)	5	1
	Placebo	5 (42%)	5	1	1	7 (58%)	3	2	9 (75%)	2	1
**Rash**	aFMT	12 (100%)	0	0	0	12 (100%)	0	0	12 (100%)	0	0
	Placebo	11 (92%)	1	0	0	11 (92%)	0	1	12 (100%)	0	0

* 0 = No adverse event (AE), 1 = Minor AE (i.e. asymptomatic), 2 = Moderate AE (symptomatic but does not impair daily life), 3 = Severe AE (very undesirable with disruptive symptoms), 4 = Life-threatening or disabling AE.

^†^ p = >0,05, no adverse events were significantly more frequent in either treatment arm.

## Discussion

There is a lack of safety and dosing data regarding autologous faecal microbiota transplantation. The primary aim of this study was therefore to evaluate the safety and tolerability of acid-resistant capsules containing autologous microbiota compared to placebo in healthy subjects. Auto-FMT given as two capsules twice daily for 5 days were well tolerated in line with previous data where enema or nasogastric tubes were used for single auto-FMT deliveries [19, 20]. Most study persons reported no adverse events during treatment with placebo or auto-FMT. Reported adverse events were primarily minor and transient and there were no significant differences between those receiving placebo or auto-FMT. Treatment with clindamycin was associated with significantly lower scores of general health and a trend towards worse intestinal health compared to treatment with auto-FMT or placebo and compared to the follow-up period. During clindamycin treatment, two study persons reported fatigue grade 3, corresponding to severe and very undesirable symptoms, for one and two days, respectively. Neither person sought medical attention for this. Thus, a total dose of 20 frozen auto-FMT capsules taken over 5 days was easy to take, safe and better tolerated than clindamycin.

The similarity of reported gastro-intestinal health, general wellbeing, and time to normalized stool pattern in the placebo and auto-FMT groups suggests that auto-FMT did not result in faster resolution of gastro-intestinal disturbance induced by clindamycin. There are several possible explanations for this. The study population was young and healthy and therefore able to rapidly and spontaneously resolve clindamycin induced gastro-intestinal disturbance. This is supported by a study where auto-FMT did not shorten time to normalized microbiota, compared with placebo, in similarly healthy study subjects in whom dysbiosis was induced by antibiotics [19]. Auto-FMT started after a two-day antibiotic-free washout period during which the microbiota most probably started to recover. In line with this, bacterial species composition and metabolic capacity had returned to baseline 7 days after end of amoxicillin-clavulanic acid treatment in the previously cited study [19]. Though the total dose of 20 capsules was similar to that used for treatment of recurrent *C*. *difficile* infection, the extended dosing regimen may have been less effective compared to a single large inoculate [12]. The extended treatment period was chosen as repeated dosing has improved efficacy when treating recurrent *C*. *difficile* infection [12]. Despite the lack of clinical difference, it is possible that the microbiota recovered faster in the group receiving auto-FMT as seen previously [20]. Future characterization of the microbiota collected as part of this study will clarify this.

The apparent safety, potential beneficial metabolic effects, ability to restore antibiotic induced dysbiosis, and low risk of pathogen spread from donors suggests that auto-FMT may become an effective and safe treatment [14, 15, 25–27]. Potential roles for auto-FMT are limited by the need to collect faecal samples prior to a risk event. Nevertheless, future use of autologous FMT include prophylactic antibiotic treatment after surgery or other interventions in populations at high risk of *C*. *difficile* infection and for the purpose of decolonization of multidrug resistant bacteria in patients undergoing cancer therapy [25, 28]. However, dose-finding and efficacy studies are needed as well as methods to effectively collect, store and administer auto-FMT. Acid-resistant capsules are certainly a convenient and, based on FMT data, an effective treatment option [13, 29].

Limitations of the study include samples size, non-observed treatment, and lack of data on stool habits prior to clindamycin treatment. The design with a questionnaire to evaluate side effects might not show us all possible events. Another limitation is that we have not been able to report microbiome analyses data that would have been of importance for our conclusions. Moreover, generalizability is affected by inviting young and healthy study subjects that contrasts with the typical FMT recipient. Nevertheless, these data are in line with previous reports [18, 19].

To conclude, the present study shows that intake of two acid-resistant capsules containing autologous microbiota twice daily for 5 days was safe and well tolerated supporting future dosing and efficacy studies.

## Supporting information

S1 Checklist Reporting checklist for randomised trial. (DOCX)

S1 File Study protocol. Study protocol for the study.
(DOCX)

S2 File Appendix B. Study questionnaires including BSC.
(DOCX)
