# Supplementary material for: Safety and tolerability of frozen, capsulized autologous faecal microbiota transplantation. A randomized double blinded phase I clinical trial
Source: PLoS One. 2023 Sep 27;18(9):e0292132. doi: 10.1371/journal.pone.0292132 (PMC10529588; doi:10.1371/journal.pone.0292132)
Supplement: S1 File — Study protocol for the study. (DOCX) [file pone.0292132.s002.docx]

**Safety and tolerability of oral, capsulized, frozen, autologous fecal microbiota inoculation after antibiotic treatment. A double blind placebo controlled study in healthy volunteers**

EudraCT number 2017-002418-30

Sponsor protocol number bactavia1

Version nr 1.0

2017-09-22

Signatures

Sponsor National coordinating investigator

____________________________ ___________________________

Table of Contents

[List of used abbreviations 4](#_Toc484611894)

[Contact information 4](#_Toc484611895)

[Summary 5](#_Toc484611896)

[1 BACKGROUND 6](#_Toc484611897)

[2. RATIONALE 7](#_Toc484611898)

[3. Cost benefit assessment 7](#_Toc484611899)

[4. AIM 8](#_Toc484611900)

[5. Outcomes 8](#_Toc484611901)

[5.1 Primary endpoint 8](#_Toc484611902)

[5.2 Secondary endpoints 8](#_Toc484611903)

[6. METHODS 8](#_Toc484611904)

[6.1 Study Design 8](#_Toc484611905)

[6.2 Study population 8](#_Toc484611906)

[6.2.1 Inclusion criteria: 8](#_Toc484611907)

[6.2.2 Exclusion criteria: 8](#_Toc484611908)

[6.3 Patient screening and patient identification logs 9](#_Toc484611909)

[6.4 RESTRICTIONS 9](#_Toc484611910)

[6.4.1 Prior and concomitant medications 9](#_Toc484611911)

[6.5 Randomization 9](#_Toc484611912)

[6.6 Blinding 9](#_Toc484611913)

[6.6.1 Breaking the treatment code (un-blinding) 10](#_Toc484611914)

[6.7 Subject withdrawal 10](#_Toc484611915)

[6.8 Preparation and storage of inoculate 10](#_Toc484611916)

[6.9 Treatment 11](#_Toc484611917)

[6.10 Monitoring and follow up: 11](#_Toc484611918)

[6.11 Laboratory Procedures 11](#_Toc484611919)

[6.11.1 Stool sampling for capsule preparation: 11](#_Toc484611920)

[6.11.2 Stool sampling for sequencing: 11](#_Toc484611921)

[6.11.3 Study of the intestinal microbiota: 12](#_Toc484611922)

[6.12 Biobank 12](#_Toc484611923)

[7. SAFETY AND ADVERSE EVENTS 12](#_Toc484611924)

[7.1 Specification of safety parameters 12](#_Toc484611925)

[7.2 Adverse event 12](#_Toc484611926)

[7.2.1 Definition 12](#_Toc484611927)

[7.3 Serious adverse event 13](#_Toc484611928)

[7.3.1 Definition 13](#_Toc484611929)

[7.4 Rating scales 13](#_Toc484611930)

[7.4.1 Intensity 13](#_Toc484611931)

[7.4.2 Causality 14](#_Toc484611932)

[7.5 Reporting procedures for serious adverse events 14](#_Toc484611933)

[7.6 Reporting period for adverse events 14](#_Toc484611934)

[7.7 Sponsor’s reporting of serious adverse drug reactions and suspected unexpected serious adverse events 14](#_Toc484611935)

[7.8 Stopping rules 15](#_Toc484611936)

[8. STUDY MANAGEMENT 15](#_Toc484611937)

[8.1 INDEPENDENT DATA SAFETY MONITORING BOARD (DSMB) 15](#_Toc484611938)

[8.2 CLINICAL MONITORING 16](#_Toc484611939)

[8.3 AUDITS AND INSPECTIONS 16](#_Toc484611940)

[8.4 TRAINING OF STUDY PERSONNEL 16](#_Toc484611941)

[8.5 CHANGES TO THE STUDY PROTOCOL 17](#_Toc484611942)

[8.6 PROTOCOL DEVIATIONS 17](#_Toc484611943)

[8.7 STUDY REPORTING 17](#_Toc484611944)

[8.8 ARCHIVING 17](#_Toc484611945)

[8.9 PATIENT CONFIDENTIALITY 17](#_Toc484611946)

[8. 10 Insurance 17](#_Toc484611947)

[8.11 PUBLICATION 17](#_Toc484611948)

[9. Ethical considerations 18](#_Toc484611949)

[11. Statistics 18](#_Toc484611950)

[11.1 Power calculation 18](#_Toc484611951)

[11.2 Duration 18](#_Toc484611952)

[11.3 Data analysis 18](#_Toc484611953)

[11.4 Dissemination of results 18](#_Toc484611954)

[12. References 19](#_Toc484611955)

# List of used abbreviations

MPA Medical Products Agency

SmPC Summary of Product Characteristics

CRF Case Record forms

IMP Investigational medical product

AE Adverse event

SAR Serious adverse event

SUSAR Suspected unexpected adverse event

# Contact information

**Sponsor:** Johan Ursing, M.D., Associate professor, Department of Infectious Diseases, Danderyd Hospital, Danderyd, Sweden

**Investigators: E-mail: Telephone:**

Johan Ursing MD, PhD [johan.ursing@gmail.com](mailto:johan.ursing@gmail.com) 0704751530

Danderyds sjukhus, Danderyd

Oscar Bladh MD [oscar.bladh@sll.se](mailto:oscar.bladh@sll.se) 0812355000 Danderyds Sjukhus, 182 88 Danderyd

Måns Stefansson, MD [mans.stefansson@gmail.com](mailto:mans.stefansson@gmail.com) 016103000

Infektionskliniken Mälarsjukhuset

63349 Eskilstuna

Hans-Peter Ekre, BSc [hans.peter.ekre@gmail.com](mailto:hans.peter.ekre@gmail.com) 0705336747

Karolinska Institutet Science Park

Fogdevreten 2A, 17165 Solna

Ola Flink, MSc. Pharm. [flink.ola@gmail.com](mailto:flink.ola@gmail.com) 0703503994

Karolinska Institutet Science Park

Fogdevreten 2A, 17165 Solna

Otto Skolling, MSc. [otto.skolling@pharmor.se](mailto:otto.skolling@pharmor.se) 0768030181

Karolinska Institutet Science Park

Fogdevreten 2A, 17165 Solna

Lars Engstrand MD, PhD, [lars.engstrand@scilifelab.se](mailto:lars.engstrand@scilifelab.se) 0706780318

Science for Life Laboratory, Solna, Sweden

Malin Nittve MSc. Pharm, MBA malin.nittve@prosaludis.com 0708368702

Pro Saludis AB, Österunda, Ytterkvarn 1,

74972 Fjärdhundra

**Monitor:**

Magnus Hedenstierna MD, PhD [magnus.hedenstierna@sll.se](mailto:magnus.hedenstierna@sll.se) 0733704540 Danderyds sjukhus, Danderyd

#

# Summary

*Clostridium difficile* is an opportunistic pathogen that causes diarrhoea and colitis and is associated with a 2.5 fold increased mortality risk. Infection typically arises following destruction of the intestinal bacterial composition (microbiota) by antibiotic treatment. Patients at risk of *C. difficile* infection can be identified through risk factors such as age and planned antibiotic exposure during e.g. planned medical interventions. *C. difficile* infection is typically treated with antibiotics. However, infection recurs in up to 30% of patients after a first episode and in up to 60% after two or more recurrences. Recurrent infections are most effectively treated by inoculating the diseased gut with bacteria from a donor (faecal microbiota transplantation). Faecal transplantation is typically a prolonged process involving family members, extensive screening and optimally gastroscopy or colonoscopy. Filtered and concentrated stool samples from unrelated donors put into capsules and frozen have recently been shown to be safe and efficacious. This study aims to assess the safety and tolerability of capsules containing autologous microbiota compared to placebo in healthy volunteers after antibiotic exposure. The time to recovery of intestinal microbiota will be determined using the next generation sequencing platform developed by Science for Life Laboratory. The long-term aim is to establish a method whereby patients can leave a stool samples prior to planned antibiotic exposure that can be used prophylactically to reconstitute intestinal microbiota whereby the risk of *C. difficile* infection is minimised and to enable rapid treatment of patients needing a faecal transplant.

# 1 BACKGROUND

*Clostridium difficile* is a toxin producing bacillus and an opportunistic pathogen that causes diarrhoea and colitis. *Clostridium difficile* infection (CDI) is a common and increasingly severe, primarily nosocomial infectious disease associated with a 2.5 fold increased 30 day mortality ([1](#_ENREF_1), [2](#_ENREF_2)). In the US there were an estimated 453 000 cases and 29 300 deaths in 2011 ([3](#_ENREF_3)). Swedish laboratories reported ~9575 episodes of CDI in Sweden in 2015 ([4](#_ENREF_4)). CDI usually occurs following antibiotic mediated destruction of the intestinal bacterial composition (microbiota) and can occur several weeks after antibiotic treatment. In line with this, the most pronounced disruption of the intestinal microbiota was during the 1^st^ month but changes lasted for at least 12 months in healthy volunteers taking clindamycin for 10 days ([5](#_ENREF_5)). Other risk factors for CDI include hospital admission, advancing age and severe underlying disease ([6](#_ENREF_6)).

A first episode of CDI is treated with metronidazole or vancomycin for 10 days. However, CDI recurs in up to 30% of patients after a first episode and in up to 60% after two or more recurrences ([7](#_ENREF_7), [8](#_ENREF_8)). The concept of treating CDI with additional antibiotics instead of reconstituting the gut flora has recently been challenged. Faecal microbiota transplantation and vancomycin treatment cured 81-94% and 23-31% of patients, respectively in a ground breaking randomised clinical trial in patients with recurrent CDI ([9](#_ENREF_9)). The effectiveness of inoculating microbiota for treatment of recurrent CDI has since been confirmed ([10-13](#_ENREF_10)). In line with this, we achieve approximately 90% treatment success when faecal microbiota transplantation, given as an enema, is used in routine clinical practice for treatment of recurrent CDI at Danderyds hospital (unpublished data). In essence faecal microbiota transplantation results in reconstitution of the normal intestinal microbiota by inoculation of a healthy individual’s microbiota into the diseased gut.

For faecal microbiota transplantation, a suitable relative is typically found and screened for various infectious entities. A fresh stool sample is collected from the donor and introduced to the patient’s intestine through a rectal enema, colonoscopy, nasogastric tube or gastroscopy. Identifying and screening takes days, enemas require that diarrhoea has been suppressed, obtaining a gastroscopy often takes time and there is a risk of regurgitation. The process thus takes time, is costly and potentially disgusting and needs to be improved.

Recent studies have shown that stools from unrelated healthy donors are as effective as stools from related donors ([14](#_ENREF_14)). Frozen stools are as effective as fresh and a preliminary feasibility study found that oral, capsulized frozen faecal microbiota transplantation cured 90% (18/20) patients with recurrent CDI ([12](#_ENREF_12), [13](#_ENREF_13)).

Antibiotic mediated destruction of the intestinal microbiota results in loss of colonization resistance not only against *C. difficile*. Drug resistant bacteria can also expand resulting in dense colonization ([15](#_ENREF_15), [16](#_ENREF_16)). This increases the risk of resistance transmission and of bacteria crossing the intestinal membranes (translocation) resulting in deep tissue and bloodstream infection ([15-17](#_ENREF_15)). Faecal microbiota transplantation rapidly reduced bacterial translocation and the presence of carbapenem resistant klebsiella in mice that had been treated with ampicillin and were infected with carbapenem resistant klebsiella ([16](#_ENREF_16)).

Replacing the donor with autologous faeces would circumvent the difficulty of finding a suitable donor and screening. Furthermore, an autologous transplant could be given prophylactically after planned antibiotic exposure such as a surgical procedure. Prophylactic reconstitution of intestinal microbiota may also reduce the risk of multidrug resistant bacteria causing infection. We therefore plan to determine the safety and tolerability of oral, capsulized, frozen autologous faecal microbiota inoculation in healthy volunteers after antibiotic treatment. The time to reconstitution of the intestinal microbiota will be assessed using the Clinical Genomics platform at the Science for Life Laboratory ([www.scilifelab.se](http://www.scilifelab.se)). This is a large scale throughput next generation sequencing based platform for the analysis of intestinal microbiota. The bacterial composition is determined by analysis of bacterial 16S rRNA gene that contains stable regions, suitable for primers and variable regions, suitable for species differentiation. More than 2000 samples have been successfully amplified using this platform and its coupled bioinformatics.

# 2. RATIONALE

Restoration of the intestinal microbiota by oral, capsulized, frozen, faecal microbiota inoculation has been shown to rapidly cure recurrent CDI. However, inoculation of donated microbiota requires extensive screening and may inoculate unwanted pathogens. Patients at high risk of *C. difficile* infection can be identified prior to planned medical procedures that further increase the risk. The long term aim of this project is to enable such patients to store faecal material for future autologous microbiota inoculation. This study specifically aims to determine the tolerability and safety of enteric resistant capsules that can be used for home based autologous microbiota inoculation. We plan to use clindamycin to disrupt the intestinal as previous data has shown the pronounced effect this drug has whilst it is also a safe commonly used and well tolerated antibiotic. Moreover, clindamycin treatment of healthy volunteers was approved by the regional ethical board in Stockholm for a study published in 2015 on the long-term effect of antibiotic administration on the human normal intestinal microbiota. The amount of bacteria in a given amount of stool varies manifold and for that reason it has not been possible to determine the dose of bacteria required to restore the intestinal microbiota. In clinical practice two spoons of faeces given as an enema typically resolve CDI. In a clinical trial 15 capsules given once daily for two days and repeated in case symptoms didn’t resolve also resolved CDI. However, giving 15 capsules in one go is not clinically practical. The proposed dosing is thus a manageable two capsules twice daily for five days which is likely to deliver a similar total amount of bacteria as the above dosing schedules.

# 3. Cost benefit assessment

The risk posed to participants are primarily allergic reactions to the antibiotic used (clindamycin) and antibiotic associated diarrhoeal disease, particularly CDI, caused by clindamycin. Clindamycin is well tolerated and rarely causes allergic reactions and the optimal treatment of CDI is inoculation with healthy intestinal microbiota which half the study participants will receive and all can do in case CDI develops. Moreover, study participants are screened for *C. difficle* prior to study entry so the risk of CDI is very low. Inoculation with intestinal microbiota has in repeated trials been shown to be well tolerated and our clinical experience is also that this treatment is well tolerated. Should anyone accidentally bite the capsule this will be disgusting but not hazardous to health. Theoretically a participant might vomit and the inhale the contents of the capsules. This is likely to cause a severe pneumonia. However, participants are healthy volunteers and the number of tablets taken at each time point is small. The risk of aspiration pneumonia is therefore exceedingly small. The potential benefit of this treatment is to develop a rapidly available treatment for CDI and for prevention of CDI, a disease that causes considerable morbidity and mortality and for which current treatment regimens are either suboptimal or very cumbersome. The benefits therefore considerably outweigh the potential risks.

#

# 4. AIM

To determine the safety and tolerability of oral, capsulized, previously frozen, autologous faecal microbiota transplantation up to 21 days after the end of faecal treatment in healthy volunteers.

# 5. Outcomes

## 5.1 Primary endpoint

The safety and tolerability of oral, capsulized, previously frozen, autologous faecal microbiota transplantation up to 21 days after the end of faecal treatment in healthy volunteers.

## 5.2 Secondary endpoints

To determine rate at which the composition of intestinal microbiota is normalised through next generation sequencing of stool samples collected for up to six months

To determine the time to normalisation of stool habits

# 6. METHODS

## 6.1 Study Design

A double blind placebo controlled phase 1 clinical trial conducted at Danderyds Hospital, Danderyd and Mälarsjukhuset, Eskilstuna both in Sweden.

The double blinded placebo controlled study design has been chosen to enable unbiased assessment of adverse events. The study will be conducted in accordance with the Helsinki declaration on ethical principals for medical research involving human subjects.

Twenty-four volunteers will be included in the study. Following informed consent participants will be requested to donate faeces. Participants will be randomised to treatment group A or B. Treatment will consist of clindamycin 300 mg three times daily for seven days for all. This will be followed by intake of two identical capsules twice daily containing placebo or an autologous faecal mixture for five days. Participants general health and gastrointestinal symptoms will be monitored using questionnaires and stool samples for sequencing of the microbiota will be collected. The study will end when the six month follow up of the last patient ends.

## 6.2 Study population

Healthy non-pregnant volunteers aged 18-40 years.

### 6.2.1 Inclusion criteria:

Written informed consent after meeting with a study physician. Ability to swallow a placebo capsule. Negative pregnancy test and use of adequate contraceptive for women while taking study treatment.

### 6.2.2 Exclusion criteria:

History of disturbed or ongoing bowel condition, delayed gastric emptying syndrome, recurrent aspirations, swallowing dysfunction, antibiotic treatment during the previous 3 months, regular intake of any medication, body mass index <18.5 and >30, any other significant medical history (except resolved traumatic injury). Identification of *C. difficile* in stool sample prior to study start.

## 6.3 Patient screening and patient identification logs

Investigators will keep a record of all patients that are considered for enrolment even if they are not subsequently enrolled in order to verify that the patient population was selected without bias (ICH-GCP 8.3.20). The reasons for non-eligibility are to be defined in terms of one or more of the eligibility criteria.

A screening number will be allocated to each patient after the subject has signed the informed consent in connection with the screening visit. The screening number will allow for the identification of patients irrespective of their possible eligibility for the main study.

## 6.4 RESTRICTIONS

Sexually active males and females of child-producing potential, must use adequate contraception (oral contraceptives, intrauterine device or barrier method of contraception in conjunction with spermicidal jelly or surgical sterile) for the duration of treatment and at least two weeks after completion of chemotherapy. Females of child-bearing potential must have a negative pregnancy test at screening for enrolment.

### 6.4.1 Prior and concomitant medications

Medication history (prior medications) is needed prior to study start. Prescription medications, over the- counter (OTC) medications, and herbal products will be asked for.

The Investigator or designee should assess changes in concomitant medications throughout the study by asking the subject at each visit. Any changes reported by the subject should be recorded in the e-CRF. Medications will be coded according to the World Health Organisation’s (WHO) Anatomic Therapeutic Chemical classification system (ATC) classification.

## 6.5 Randomization

Participants will be concurrently randomly allocated into two arms in a 1:1 fashion. Randomization is to be performed after eligibility has been confirmed and at least 1 hour prior to start of clindamycin treatment.

Patients who fulfil all the inclusion criteria and none of the exclusion criteria are eligible for inclusion into the study and will be randomised into two arms:

- Group A: Clindamycin + Capsules with faeces solution
- Group B: Clindamycin + Placebo Capsules

Randomization is performed centrally.

## 6.6 Blinding

Identical capsules will be given to the subjects. After randomization, the two groups of subjects are followed in exactly the same way. Treatment code envelopes will be provided for each randomised subject.

### 6.6.1 Breaking the treatment code (un-blinding)

Treatment code envelopes will be provided for each randomised subject. The code envelopes will be kept in a secure place with limited access. In case of such emergency that it is crucial for the Investigator, or any other treating physician, to know whether the subject has received study product or placebo, the code envelope may be opened. If the code is broken, this must be documented on the treatment code envelope and in the subject’s hospital records with date and name of the Investigator who decided to break the code.

In the event of an SAE, the Investigator may only break the treatment code if the appropriate future management of the subject necessitates knowledge of the current treatment. Although it is advantageous to retain the blind for all subjects prior to final study analysis, when a SAE may be a serious adverse reaction unexpected or otherwise judged reportable on an expedited basis, it is recommended that the blind should be broken only for that specific subject, by the sponsor, even if the Investigator has not broken the blinding.

The treatment code will not be broken until all assessments have been performed, all data have been entered into the database and the database has been locked after a formal clean file has been produced. The safety committee will monitor data in a blinded fashion during the study.

## 6.7 Subject withdrawal

Subjects are free to discontinue their participation in the study at any time and for whatever reason without affecting their right to an appropriate follow-up investigation. If possible, the reason for withdrawal of consent should be documented.

Once a subject has been included in the study, the Investigator should make every reasonable effort to keep the subject in the study.

Subjects may be discontinued from the study at any time at the discretion of the Investigator. Specific reasons for discontinuing a subject from further assessments are:

- Withdrawal of informed consent
- Subject lost to follow-up (i.e. drop-outs)
- Death
- Substantial Clinical Study Protocol violation
- The Investigator’s decision

The Investigator will make every reasonable effort to complete the procedures outlined for the end of treatment visit for subjects who have received study medication and are subsequently withdrawn from the study.

##

## 6.8 Preparation and storage of inoculate

Following inclusion volunteers will be requested to leave a faecal sample. The faecal sample will be left at Danderyds hospital or Mälarsjukhuset, Eskilstuna.

The faeces will be homogenized, filtered and glycerol added before freezing. The faeces will be thawed prior to capsulation and a surfactant and vegetable oil added. Capsules will be filled with this inoculate.

## 6.9 Treatment

Participants will come to the Department of Infectious Diseases, Danderyds hospital or Mälarsjukhuset in Eskilstuna the day prior to treatment start to collect clindamycin and for a final briefing. All participants will be requested to take 300 mg clindamycin capsules morning, lunch and evening for seven days. This will be followed by a two and a half day washout period. On day three of the washout period participants will return to Hospital to take the first IMP or placebo dose supervised and to collect their remaining study capsules that are to be stored in a freezer at home. Participants will then be requested to take two study capsules twice daily for a total of five days.

An SMS text message will be sent twice daily to remind volunteers to take the clindamycin and study capsules. Patients and investigators will be blinded as to the content of the capsules. The code will be held by the manufacturers of the capsules that are not involved in the clinical part of the study.

## 6.10 Monitoring and follow up:

Upon inclusion clinical records form (appendix A) will be completed.

Stool habits and characteristics will be monitored daily from treatment start until day 28 and at 2 and 6 months using the structured questionnaire in appendix B. Questionnaires record stool frequency and consistency, general and gastrointestinal well-being via a standardized health score, rating of gastrointestinal symptoms and medication use. Overall and gastrointestinal-specific health scores will be reported on a scale of 1 to 10, with 1 being the lowest and 10 being “best possible health for you.”

Stool samples for sequencing will be taken at baseline and on days 10, 16 (+3 days if necessary), 28 and at 2 and 6 months.

**Table 1. Outline of study and sampling**

| Days |  | 1 | 1-7 | 8-10 | 10-15 | 16 | 28 | 2 months | 6 months |
| --- | --- | --- | --- | --- | --- | --- | --- | --- | --- |
| Phase | Inclusion | Start | Antibiotic | Washout | Treat | Follow up | | | |
| CRF A | + |  |  |  |  |  |  |  |  |
| *C.diff* screening | + |  |  |  |  |  |  |  |  |
| Faecal sample* | + | + |  | + |  | + | + | + | + |
| CRF B |  | + | + | + | + | + | + | + | + |
| Hospital visit | + | + |  | + |  | + |  |  |  |

*Prior to study start a faecal sample for autologous transplant will be donated.

## 6.11 Laboratory Procedures

### 6.11.1 Stool sampling for capsule preparation and sequencing:

A same day stool sample (minimum volume approximately 2 table spoons full) stored in a dedicated container will be delivered to Danderyds Hospital or Mälarsjukhuset as outlined in Table 1. The sample will then be transported to Reagensia AB, Solna and to SciLife Lab, Solna for capsule preparation and analyses of bacterial composition (microbiota).

### 6.11.2 Study of the intestinal microbiota:

Massive parallel sequencing will be performed. The patient will take a stool sample (approximate one spoon in dedicated device) immediately after defecation and introduce it into a transport medium for subsequent microbiota analyses in a sterile tube. Samples will be sent by ordinary mail (using standard shipping procedures) to the hospital laboratory and frozen upon arrival at minus 70 0C

Sequencing libraries for microbiota profiling will be prepared by amplifying the V3-V4 region of the 16S rRNA gene. After the initial amplification a second PCR will be performed to attach Illumina adapters as well as barcodes that allows for multiplexing. Samples will be sequenced using the Illumina MiSeq, whereafter primer sequences will trimmed away and the paired-end reads produced by the sequencing instrument will be merged using SeqPrep verson 1.1 (<https://github.com/jstjohn/SeqPrep>) with default parameters and thereafter processed with the QIIME 1.8.0 pipeline (Quantitative Insight Into Microbial Ecology). Using this pipeline, sequences will be clustered at 97% identity against the Greengenes reference database ([18](#_ENREF_18)).

## 6.12 Biobank

Stool samples and blood samples will be stored as part of the Karolinska Institutet biobank.

# 7. SAFETY AND ADVERSE EVENTS

## 7.1 Specification of safety parameters

Safety parameters; Adverse drug reactions and serious adverse drug reactions and changes in vital signs.

Any apparent side effects experienced by the subject will be assessed from the time the subject signs the informed consent and throughout the course of the entire study, and will be reported by study site personnel either as a Baseline Event or an AE (6.2). The study personnel will document any Baseline Events or AEs in the CRF, whether observed by the Investigator or reported by the subject. The safety of the different treatment arms will be assessed with regard to AEs, baseline medical conditions, and findings from the physical examination and laboratory tests.

Possible adverse events will be elicited using a modification of the Common Terminology Criteria for Adverse Events version 3.0. as used by Youngster et al ([13](#_ENREF_13), [19](#_ENREF_19)). Fever, gastrointestinal symptoms, headache, fatigue, and rash will be the principle symptoms evaluated (appendix B).

The AE reporting period is described in Section 6.2.

## 7.2 Adverse event

### 7.2.1 Definition

An AE in this study is defined as any untoward medical occurrence in a subject who has received investigational medicinal product (IMP). The occurrence does not necessarily need to have a causal relationship with the IMP. An AE can therefore be any unfavourable and unintended sign (including an abnormal laboratory finding), symptom, or disease temporally associated with the administration of IMP, whether or not causally related.

The occurrence of an AE may come to the attention of study personnel during study visits and interviews of a study recipient presenting for medical care, or upon review by a study monitor who is scrutinising relevant source data.

Clarifications:

Diagnostic and therapeutic non-invasive and invasive procedures, such as surgery, should not be reported as AEs. However, the medical condition for which the procedure was performed should be reported if it meets the definition of an AE. For example, an acute appendicitis that begins during the AE reporting period should be reported as “acute appendicitis” and the resulting appendectomy noticed under Comments. Pre-study conditions, which led to elective surgery during the time of the study, are not to be reported as AEs.

If an abnormal laboratory value or vital sign is associated with corresponding clinical signs and symptoms, the sign/symptom should be reported as the AE and the associated laboratory result or vital sign should be considered additional information that is to be collected in the CRF.

## 7.3 Serious adverse event

### 7.3.1 Definition

An SAE in this study is defined as any untoward medical occurrence that meets one of the following criteria:

- Results in death
- Is life threatening (the term “life threatening in the definition of “serious” refers to an event in which the subject was at risk of death and the time of the event; it does not refer to an event which hypothetically might have caused death if it was more severe)
- Requires inpatient hospitalization or prolongation of existing hospitalization
- Results in persistent or significant disability/incapacity
- Is an important medical event

## 7.4 Rating scales

The Investigator is to record all directly observed AEs and all AEs spontaneously reported by the subject in the subject’s records (source data) and in the CRFs using concise medical diagnostic terminology. All AEs must be graded for:

- seriousness
- intensity
- causality (possible relationship) to the IMP

The question asked will be “Have you had any health problems since your last evaluation?” If no AE has occurred during the period concerned, this should actively be noted in the CRF.

### 7.4.1 Intensity

The expression intensity of adverse events means the intensity of the event in the opinion of the subject. The intensity of each AE is to be graded by the Investigator.

### 7.4.2 Causality

The relationship between the IMP and each AE has to be classified by the Investigator using one of the following terms:

Unlikely: The onset of the AE and administration of the IMP are such that the medication is not likely to have any reasonable association with the AE.

Possible: It might be possible that the AE could have been caused by the IMP.

Probable: It is probable that the AE is caused by the IMP.

## 7.5 Reporting procedures for serious adverse events

Events deemed as serious (see Section 7.3) must be reported to the investigator within 24 hours after the Investigator’s awareness of the SAE. This short time frame is in compliance with international regulations. All SAEs should be reported by the site staff in the CRF.

If the initial report is not complete, it should be followed by submission of a more detailed report within five calendar days.

All SAEs must be followed until resolution or until the Investigator assesses them as being under full control.

The reporting period for SAEs starts at capsule administration and ends at the final follow-up visit 6 months after end of IMP administration.

## 7.6 Reporting period for adverse events

All AEs, irrespective of nature, will be followed until resolution or until end of the follow-up period.

The reporting period for AEs starts at capsule administration and ends at the final follow-up visit 6 months after end of IMP administration.

## 7.7 Sponsor’s reporting of serious adverse drug reactions and suspected unexpected serious adverse events

The Investigator and /or Bactavia or designee have to make a causality (relationship) assessment. The term SADR (Serious Adverse Drug Reaction) is to be used when the Investigator deems the SAE as possibly or probably related to the IMP. Serious Adverse Drug Reactions are to be reported annually to the MPA and the ethical committee.

If the event is not described before, e.g*.* in the IB, the event is a SUSAR (Suspected Unexpected Serious Adverse Event). Bactavia or designee has the obligation to submit SUSAR reports electronically to the Eudravigilance database within:

- 7 days if fatal or life-threatening (follow-up information within an additional 8 days)
- 15 days if non-fatal and non-life-threatening (follow-up information as soon as possible)

The following bodies are recipients of the reports, and procedures for such reports are familiar to investigator or designee:

- MPA
- European Medicines Agency (EMA)
- The study approving IEC

Bactavia has delegated the task of reporting SADR/SUSARs to the investigator, but has the ultimate responsibility for the fulfilment of these tasks. This delegation is enforced in a written delegation.

Bactavia also has the obligation to, once a year throughout the clinical study (or on request); submit a safety report to the MPA and the IEC taking into account all new available safety information received during the reporting period.

## 7.8 Stopping rules

If an attending physician assesses that a patient is suffering from an adverse event induced by the IMP and requiring treatment, IMP treatment will be stopped for that patient. The outcome will be classified as an adverse event necessitating treatment stop. Should a study participant develop CDI this will be treated as per routine with metronidazole and in case of recurrence with autologous faecal transplant as per routine.

The data and safety monitoring board and the Swedish Medical Products Agency have the right to stop the study.

A study subject can terminate his/her participation in the study at any time without giving a reason why as outlined in patient information (appendix C). Should a subject wish to do so he/she will be treated in line with standard recommendations.

Study organisers can end a patient’s participation for safety reasons.

If a patient is excluded, already collected data will be used in subsequent analyses unless he/she objects.

# 8. STUDY MANAGEMENT

## 8.1 INDEPENDENT DATA SAFETY MONITORING BOARD (DSMB)

An independent Data Safety and Monitoring Board is required if a study poses a considerable risk to study participants and if the study duration is long and divided into blinded and nonblinded groups. Oral capsules with faecal bacteria from healthy donors have been shown to be safe and well tolerated in several clinical trials ([13](#_ENREF_13), [20](#_ENREF_20), [21](#_ENREF_21)). We are using the same study capsule as in previous studies but with autologous faeces. Furthermore, the dosing protocol is two capsules twice daily for five days compared to 15 capsules once daily for two days in previous studies. If anything, this schedule is likely to result in fewer adverse events as intake of 15 capsules in one go is potentially difficult. The follow up is only 6 months and the healthy patients are studied. A data safety and monitoring board is therefore not necessary.

## 8.2 CLINICAL MONITORING

Before the initiation of the study, the monitor will:

- Determine the adequacy of the facilities
- Discuss with the Investigator and study personnel their responsibilities with regard to CSP adherence, local regulations, and the duties of monitor
- At an initiation meeting, the monitor will comply with the ICH-GCP Guidelines (ICH-GCP 8.3.20) and document that the study procedures were reviewed with the Investigator and the Investigator’s staff
- During the study, a monitor will pay visits to the investigational site in order to:
  - provide information and support to the Investigator
  - confirm that facilities remain acceptable
  - confirm that the investigational team is adhering to the CSP
  - confirm that data are being accurately recorded in the e-CRFs
  - ensure that accountability checks for the IMP are being performed
  - conduct source data verification, which will require direct access to all original records for each subject (e.g. medical records)

The monitor will be available (by phone, fax and e-mail) between visits whenever the Investigator or other study personnel at the investigational site needs information, advice or help.

All documentation and correspondence pertaining to the study (raw data, letters etc.) should be kept in accordance with ICH-GCP.

## 8.3 AUDITS AND INSPECTIONS

The purpose of an audit or inspection is to systematically and independently examine all study related activities to document that they were conducted, recorded, analyzed and accurately reported according to the CSP and the background regulatory demands.

Audits or inspections may therefore be performed at the study site during or after the study. Visits may thereby be paid by the MPA. These visits may include source data verification and confidentiality documents are therefore created.

The Investigator should contact the monitor immediately if they are contacted by the MPA about an inspection at their study site.

## 8.4 TRAINING OF STUDY PERSONNEL

The Investigator will maintain records of all individuals involved in the study (medical, nursing and other personnel). The Investigator will ensure that appropriate training relevant to the study is given to the personnel involved in the study, and that any new information of relevance to the conduct of the study is forwarded to the persons involved.

## 8.5 CHANGES TO THE STUDY PROTOCOL

Study procedures will not be changed without mutual agreement between the Investigator and Bactavia.

If the clinical study protocol (CSP) needs to be amended, the amendment or a new version of the CSP must be approved by the MPA and the ethical committee before implementation. Approval must also be obtained for the written Patient Information and Informed Consent Form, if applicable.

If an amendment to the CSP substantially alters the study design, or increases the potential risk to the patients, written informed consent must be obtained again for currently enrolled patients and must be provided to additional patients prior to their entry into the study.

## 8.6 PROTOCOL DEVIATIONS

Deviations from the CSP, deemed necessary for an individual subject, will be reported in the e-CRF giving the reason and date. If necessary, the Investigator (or designee) will contact the monitor to inform about the deviation.

## 8.7 STUDY REPORTING

After completion of the study, the Investigator will prepare a clinical study report in co-operation with Bactavia. The Investigator is responsible for submitting the final study report to the MPA.

## 8.8 ARCHIVING

The Investigator shall keep records of the study for 10 years after final signed 12-month Clinical Study Report. This includes any original source documents related to the study, including the subject Identification List with Subject Numbers, full names and the original signed informed consent forms and detailed records of disposition of IMP.

## 8.9 PATIENT CONFIDENTIALITY

The patients have the right to request access to his/her personal data and the right to request rectification of any data that is not correct and/or complete. Investigator or designee personnel whose responsibilities require access to personal data agree to keep the identity of each patient confidential. This agreement is to be substantiated in a separate document.

## 8. 10 Insurance

Participants are covered by “patientskadelagen” and the investigational medicinal product betamethasone is covered by “läkemedelsförsäkringen”.

## 8.11 PUBLICATION

The Investigator is responsible for registering the study in a publicly accessible database before recruitment of the first patient.

The study report (Section 8.7) may form the basis for a manuscript intended for publication in a medical journal. Attempts to publish negative or inconclusive as well as positive results must be made, or otherwise made publicly available.

#

# 9. Ethical considerations

The trial has been registered with the EU’s electronic database of clinical trials (EudraCT), with EudraCT number 2017-002418-30, and sponsor protocol number bactavia1. The trial will be submitted to the Swedish Medical Products Agency (läkemedelsverket) and Regional Ethical Review Board in Stockholm for review. Following approval the study will be started. Patients will be informed of the study verbally and in writing. Details about the trial including risks and benefits will be explained and any questions answered. A copy of the written consent form (appendix C) will then be signed. The use of clindamycin in healthy individuals to study the effect on the intestinal microbiota without access to autologous faecal transplantation was approved by the Stockholm regional ethical board in a recent study ([5](#_ENREF_5)).

**10**. QUALITY CONTROL (QC) AND QUALITY ASSURANCE (QA)

This study will be conducted in compliance with this protocol, standard operating procedures at the study site, the ICH Guideline for GCP and any local regulations.

Audit of the study sites may be conducted to assess and help assure compliance with GCP and applicable regulatory requirements. The study sites may be subject to a QA audit by the sponsor or its representatives, as well as this study may be reviewed by an independent QA department and/or inspected by regulatory authorities. This implies that auditors/inspectors will have the right to inspect the study sites at any time during and/or after completion of the study and will have access to source documents, including patients’ medical records. By participating in this study, the investigator agrees to this requirement.

# 11. Statistics

## 11.1 Power calculation

No power calculation was done for this safety and tolerability study. Twelve patients in each arm are considered sufficient to detect major adverse events whilst avoiding unnecessary exposures.

## 11.2 Duration

Advertising and recruitment will be done during autumn 2017. The study should start around November/December 2017 and run for 6 months. Sequencing and data analysis will take 6 months. A report should be completed by the end of 2018. Within 90 days of the end of the study the “declaration of end of trial notification” document will be sent to the MPA

## 11.3 Data analysis

Final analysis will include a description of included participants, proportions of adverse events and any serious adverse events, the proportion of participants withdrawn or lost to follow up. Categorical variables will be compared between patient groups using the χ^2^ test or Fisher exact test and continuous variables using quantile regression.

For microbiota Shannon indexes for diversity will be calculated in all samples and tested for significance with Wilcoxon rank-sum test. Using the QIIME pipeline, unweighted UniFrac distances are then produced and used for investigation of beta diversity through plotting PCA coordinates

## 11.4 Dissemination of results

Results will be published in an international peer reviewed journal

#

# 12. References

1. Bartlett JG. Clinical practice. Antibiotic-associated diarrhea. N Engl J Med. 2002;346(5):334-9.

2. Hensgens MP, Goorhuis A, Dekkers OM, van Benthem BH, Kuijper EJ. All-cause and disease-specific mortality in hospitalized patients with Clostridium difficile infection: a multicenter cohort study. Clin Infect Dis. 2013;56(8):1108-16.

3. Lessa FC, Mu Y, Bamberg WM, Beldavs ZG, Dumyati GK, Dunn JR, et al. Burden of Clostridium difficile infection in the United States. N Engl J Med. 2015;372(9):825-34.

4. Mäkitalo B, Åkerlund T. Clostridium difficile rapport: Folkhälsomyndigheten; 2016 [Available from: <http://www.folkhalsomyndigheten.se/amnesomraden/statistik-och-undersokningar/sjukdomsstatistik/clostridium-difficile-infektion/>.

5. Rashid MU, Zaura E, Buijs MJ, Keijser BJ, Crielaard W, Nord CE, et al. Determining the Long-term Effect of Antibiotic Administration on the Human Normal Intestinal Microbiota Using Culture and Pyrosequencing Methods. Clin Infect Dis. 2015;60 Suppl 2:S77-84.

6. Garey KW, Sethi S, Yadav Y, DuPont HL. Meta-analysis to assess risk factors for recurrent Clostridium difficile infection. J Hosp Infect. 2008;70(4):298-304.

7. Pepin J, Valiquette L, Gagnon S, Routhier S, Brazeau I. Outcomes of Clostridium difficile-associated disease treated with metronidazole or vancomycin before and after the emergence of NAP1/027. Am J Gastroenterol. 2007;102(12):2781-8.

8. Vardakas KZ, Polyzos KA, Patouni K, Rafailidis PI, Samonis G, Falagas ME. Treatment failure and recurrence of Clostridium difficile infection following treatment with vancomycin or metronidazole: a systematic review of the evidence. Int J Antimicrob Agents. 2012;40(1):1-8.

9. van Nood E, Vrieze A, Nieuwdorp M, Fuentes S, Zoetendal EG, de Vos WM, et al. Duodenal infusion of donor feces for recurrent Clostridium difficile. N Engl J Med. 2013;368(5):407-15.

10. Costello SP, Conlon MA, Vuaran MS, Roberts-Thomson IC, Andrews JM. Faecal microbiota transplant for recurrent Clostridium difficile infection using long-term frozen stool is effective: clinical efficacy and bacterial viability data. Aliment Pharmacol Ther. 2015;42(8):1011-8.

11. Kelly CR, Ihunnah C, Fischer M, Khoruts A, Surawicz C, Afzali A, et al. Fecal microbiota transplant for treatment of Clostridium difficile infection in immunocompromised patients. Am J Gastroenterol. 2014;109(7):1065-71.

12. Lee CH, Steiner T, Petrof EO, Smieja M, Roscoe D, Nematallah A, et al. Frozen vs Fresh Fecal Microbiota Transplantation and Clinical Resolution of Diarrhea in Patients With Recurrent Clostridium difficile Infection: A Randomized Clinical Trial. Jama. 2016;315(2):142-9.

13. Youngster I, Russell GH, Pindar C, Ziv-Baran T, Sauk J, Hohmann EL. Oral, capsulized, frozen fecal microbiota transplantation for relapsing Clostridium difficile infection. Jama. 2014;312(17):1772-8.

14. Youngster I, Sauk J, Pindar C, Wilson RG, Kaplan JL, Smith MB, et al. Fecal microbiota transplant for relapsing Clostridium difficile infection using a frozen inoculum from unrelated donors: a randomized, open-label, controlled pilot study. Clin Infect Dis. 2014;58(11):1515-22.

15. Blot S, Depuydt P, Vogelaers D, Decruyenaere J, De Waele J, Hoste E, et al. Colonization status and appropriate antibiotic therapy for nosocomial bacteremia caused by antibiotic-resistant gram-negative bacteria in an intensive care unit. Infect Control Hosp Epidemiol. 2005;26(6):575-9.

16. Caballero S, Carter R, Ke X, Susac B, Leiner IM, Kim GJ, et al. Distinct but Spatially Overlapping Intestinal Niches for Vancomycin-Resistant Enterococcus faecium and Carbapenem-Resistant Klebsiella pneumoniae. PLoS Pathog. 2015;11(9):e1005132.

17. Donskey CJ. The role of the intestinal tract as a reservoir and source for transmission of nosocomial pathogens. Clin Infect Dis. 2004;39(2):219-26.

18. Caporaso JG, Kuczynski J, Stombaugh J, Bittinger K, Bushman FD, Costello EK, et al. QIIME allows analysis of high-throughput community sequencing data. Nat Methods. 2010;7(5):335-6.

19. Trotti A, Colevas AD, Setser A, Rusch V, Jaques D, Budach V, et al. CTCAE v3.0: development of a comprehensive grading system for the adverse effects of cancer treatment. Semin Radiat Oncol. 2003;13(3):176-81.

20. Hirsch BE, Saraiya N, Poeth K, Schwartz RM, Epstein ME, Honig G. Effectiveness of fecal-derived microbiota transfer using orally administered capsules for recurrent Clostridium difficile infection. BMC Infect Dis. 2015;15:191.

21. Youngster I, Mahabamunuge J, Systrom HK, Sauk J, Khalili H, Levin J, et al. Oral, frozen fecal microbiota transplant (FMT) capsules for recurrent Clostridium difficile infection. BMC Med. 2016;14(1):134.
