# Supplementary material for: Safety and tolerability of frozen, capsulized autologous faecal microbiota transplantation. A randomized double blinded phase I clinical trial
Source: PLoS One. 2023 Sep 27;18(9):e0292132. doi: 10.1371/journal.pone.0292132 (PMC10529588; doi:10.1371/journal.pone.0292132)

Kliniskt studie formulär för dagarna 1-28 och efter 2 och 6 månader

Pnr________________________________ Namn____________________________

Studie ID_____________________________ Inklusionsdatum___________________

Formen ifylld av_______________________ Dagens datum______________________

Behandlings-, uppföljningsdag______

Tarmtömningar sista dygnet

| Tarmtömning | Bristol typ 24 hrs |
| --- | --- |
| 1 |  |
| 2 |  |
| 3 |  |
| 4 |  |
| 5 |  |

Kroppstemperatur (morgonen) _____

Nya mediciner (antibiotika?) 1. ____________________________

2._____________________________

Allmän hälsoskala (hur mår du?): SÄMST = 1 2 3 4 5 6 7 8 9 10 = BÄST

Mage/tarm hälsoskala (hur är magen/tarmen?): SÄMST = 1 2 3 4 5 6 7 8 9 10 = BÄST

Vänligen fyll nu i specifika frågor om hur du mår på nästa sida enligt skalan som finns på sidan efter det.

Graderingstabell för biverkningar/symptom

|  | 0 (inga besvär) | 1 (mild) | 2 (måttlig) | 3 (allvarlig) | 4 (potentiellt livshotande) |
| --- | --- | --- | --- | --- | --- |
| Diarré |  |  |  |  |  |
| Illamående |  |  |  |  |  |
| Trötthet/sjukdomskänsla |  |  |  |  |  |
| Kräkningar |  |  |  |  |  |
| Huvudvärk |  |  |  |  |  |
| Uppblåst buk |  |  |  |  |  |
| Buksmärta |  |  |  |  |  |
| Yrsel |  |  |  |  |  |
| Utslag |  |  |  |  |  |
| Sväljningssvårigheter |  |  |  |  |  |

Har du haft några andra hälsoproblem sedan föregående evaluering?

___________________________________________________________________________

___________________________________________________________________________

___________________________________________________________________________

Ytterligare kommentarer

___________________________________________________________________________

___________________________________________________________________________

___________________________________________________________________________

___________________________________________________________________________

___________________________________________________________________________

___________________________________________________________________________

|  | Mild – 1 | Måttlig – 2 | Allvarlig – 3 | Potentiellt livshotande - 4 |
| --- | --- | --- | --- | --- |
| Svårighets grad – Allmänt | |  |  |  |
|  | Symptomen har ingen eller minimal påverkan på det dagliga livet | Symptomen har mer än minimal påverkan på det dagliga livet | Symptomen förhindrar aspekter av det dagliga livet | Symptomen förhindrar basal omvårdnad eller leder till medicinsk eller operativ intervention för att förhindra permanent skada |
| Symptom specifik svårighetsgrad | |  |  |  |
| Feber | 38-39 | >39-40 | >40 | >40 i >24 timmar |
| Diarré | Övergående episod eller en ökning med ≤3 avföringar per dygn | Ihållande diarréer eller en ökning med 4-6 avföringar per dygn | Blodig diarré eller en ökning med ≥7 avföringar per dygn eller behov av IV vätskor | Livshotande, eg hypotensiv shock |
| Illamående | Övergående <24 timmar eller intermittent illamående | Ihållande illamående som orsakar minskat intag per os i 24-48 timmar | Ihållande illamående som orsakar minimalt intag per os i >48 timmar eller som kräver IV vätskor | Livshotande |
| Kräkningar | Övergående eller intermittenta kräkningar med endast minimal påverkan på intag per os | Frekventa kräkningar med lätt dehydrering | Ihållande kräkningar med ortostatisk hypotension eller behov av IV vätskor | Livshotande |
| Trötthet, sjukdomskänsla | Symptomen har ingen eller minimal påverkan på det dagliga livet | Symptomen har mer än minimal påverkan på det dagliga livet | Symptomen förhindrar aspekter av det dagliga livet | Förlamande trötthet som orsakar oförmåga att klara vardaglig omvårdnad |
| Huvudvärk | Symptomen har ingen eller minimal påverkan på det dagliga livet | Symptomen har mer än minimal påverkan på det dagliga livet | Symptomen förhindrar aspekter av det dagliga livet | Huvudvärken orsakar oförmåga att klara vardaglig omvårdnad eller hämmar neurologiska eller kognitiva funktioner påtagligt |


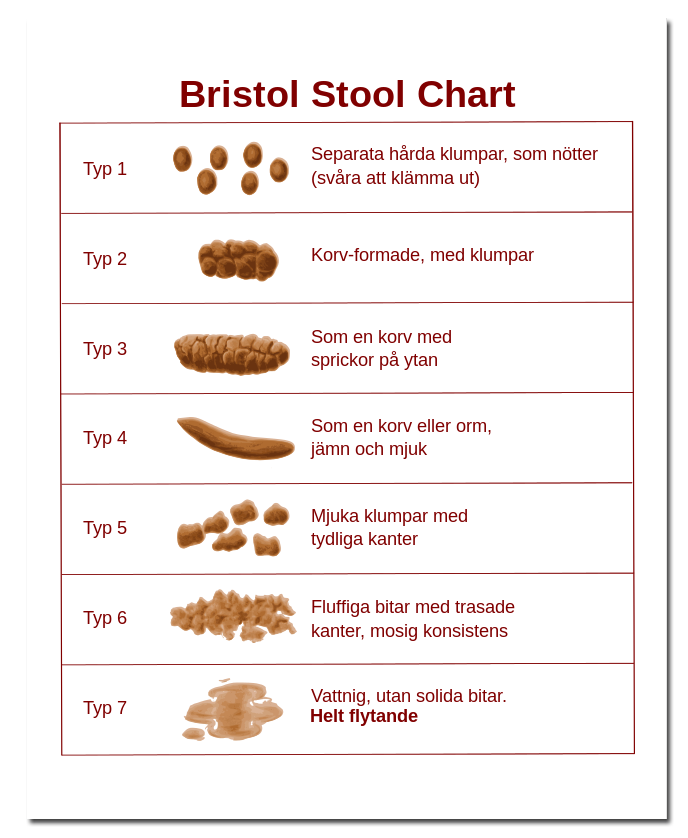

Supplement: S2 File — Study questionnaires including BSC. (DOCX) [file pone.0292132.s003.docx]
